# Diagnostic performance of two molecular assays for the detection of vaginitis in symptomatic women

**DOI:** 10.1007/s10096-019-03694-w

**Published:** 2019-09-09

**Authors:** Alexandra Thompson, Karen Timm, Noelle Borders, Liz Montoya, Karissa Culbreath

**Affiliations:** 1 University of New Mexico School of Medicine, Albuquerque, NM USA; 2 TriCore Reference Laboratories, Albuquerque, NM USA; 3 Department of Obstetrics and Gynecology, University of New Mexico School of Medicine, Albuquerque, NM USA

**Keywords:** Vaginitis, *Trichomonas*, Sexually transmitted infection, Molecular diagnostics

## Abstract

The three main causes of vaginitis are bacterial vaginosis (BV), vulvovaginal candidiasis (VVC), and trichomoniasis (TV). Two multiplex assays are commercially available for detection of DNA from organisms associated with vaginitis: BD Affirm™ VPIII Microbial Identification Test (Affirm) and BD MAX™ Vaginal Panel (MAX VP). Here, the performance of MAX VP was compared to that of Affirm, which was considered the standard of care. Four vaginal swabs were collected from each subject with the following: BD Affirm™ VPIII Ambient Temperature Transport System (ATTS), BD MAX™ UVE Specimen Collection Kit, Hologic Aptima® Vaginal Swab Specimen Collection Kit, and BD ESwab™ collection and transport system (ESwab). *Candida* culture, Gram stain followed by Nugent scoring, and the Hologic Aptima® *Trichomonas vaginalis* assay were used for discordant analysis. Results were considered true positive if there were at least two tests positive for any vaginitis target. A total of 200 symptomatic women were evaluated in the study. The sensitivity and specificity of MAX VP for BV was 96.2% and 96.1%, respectively, compared to 96.2% and 81.6% for Affirm. The sensitivity and specificity of MAX VP for *Candida* spp. was 98.4% and 95.4%, respectively, compared to 69.4% and 100% for Affirm. MAX VP and Affirm showed 100% concordance for detection of TV. These results demonstrate improved accuracy of MAX VP compared to Affirm for the detection of BV and *Candida* spp. and no difference for detection of TV between the two tests.

## Introduction

Vaginitis encompasses a spectrum of conditions that cause vaginal and vulvar symptoms, including thickened or malodorous discharge, dysuria, itching, and general discomfort [1]. These complaints are the cause of ten million office visits per year [2]. Vulvovaginal candidiasis (VVC), trichomoniasis (TV), and bacterial vaginosis (BV) are three distinct infectious syndromes collectively classified as vaginitis causes [1]. VVC involves fungal infections of the vagina and is most commonly caused by *Candida albicans*; however, other species including *C. tropicalis* and *C. glabrata* are also implicated in VVC [3]. TV is a sexually transmitted infection caused by the flagellated protozoan *Trichomonas vaginalis*, which infects the vaginal epithelium [4]. The etiology of BV continues to evolve; however, symptomatic infection is generally associated with an imbalance in the vaginal flora resulting from a decrease in one or more species of the *Lactobacillus* genus with a concomitant increase of BV-associated bacteria including *Gardnerella vaginalis*, *Atopobium vaginae*, or other anaerobic bacteria [5, 6].

Accurate diagnosis and management of vaginitis is challenging due to the nonspecific nature of patient-reported symptoms, which may overlap with multiple etiologic agents that do not consistently predict the underlying cause(s) [7]. In-clinic diagnostic methods lack sensitivity and specificity for the detection and differentiation of causative agents of vaginitis [8]. Recurrent infections are common, requiring multiple clinic visits, which may be attributed to initial misdiagnosis of these syndromes [9]. Treatments for these conditions are different, which can contribute to repeat visit(s) if the initial diagnosis is incorrect [10]. Although morbidity may be low, there are significant risks associated with misdiagnosis of vaginitis. BV and TV have been linked to adverse pregnancy outcomes (preterm labor and premature rupture of membranes), pelvic inflammatory disease, and increased risk of transmission and acquisition of STIs, including HIV [11–14]. Therefore, careful history and examination, combined with accurate testing, is essential for a correct diagnosis and appropriate treatment course for patients [1].

The BD Affirm™ VPIII Microbial Identification Test (Affirm, Becton, Dickinson and Company, Sparks, MD) is a DNA probe hybridization test used to detect *Gardnerella vaginalis*, *Candida* spp. (including *C. albicans*, *C. glabrata*, *C. kefyr*, *C. krusei*, *C. parapsilosis*, and *C. tropicalis*), and *T. vaginalis* [15]. BD MAX™ Vaginal Panel (MAX VP; Becton, Dickinson and Company, Quebec, Canada) uses real-time PCR for the amplification of specific DNA targets to differentially detect (a) bacterial vaginosis through algorithmic analysis of lactobacilli (*L. crispatus* and *L. jensenii*) and bacteria involved in BV (*G. vaginalis*, *A. vaginae*, *Megasphaera*-1, and BVAB-2); (b) *Candida* group (*C. albicans*, *C. tropicalis*, *C. parapsilosis*, and *C. dubliniensis*), *C. glabrata*, and *C. krusei*; and (c) *Trichomonas vaginalis*. Previous studies have described the performance of MAX VP compared to clinical diagnosis and nonamplification methods [8, 16]. In this study, we performed a clinical evaluation of the performance of MAX VP compared to Affirm, the standard of care, for the detection of putative vaginal pathogens in symptomatic women.

## Methods

### Study design

A total of 215 symptomatic women presented to four obstetrics and gynecology clinics in the University of New Mexico Health Systems and consented to study participation. Patients were excluded from the study if they did not meet the inclusion criteria or did not consent to participate. Of the 215 women enrolled, 15 were excluded (10 duplicate patients; 1 incorrect specimen collection; 2 consent forms not completed; 2 unable to provide a specimen). The study was conducted under the approval of the Institution Review Board of the University of New Mexico.

### Sample collection and vaginitis assays and patient clinical data

A total of four vaginal swab specimens per patient were collected for the study. These specimens included (1) BD Affirm™ VPIII Ambient Temperature Transport System (Becton, Dickinson and Company, Sparks, MD), (2) BD MAX™ UVE Specimen Collection Kit (Becton, Dickinson and Company, Sparks, MD), (3) Hologic Aptima® Vaginal Swab Collection Kit (Hologic Inc., San Diego, CA), and (4) BD ESwab™ collection and transport system (ESwab; Becton, Dickinson and Company, Sparks, MD). All swabs were transported at 2–4 °C. All swabs and transport systems were used according to the manufacturers’ instructions. Affirm, MAX VP, and the Hologic Aptima® *Trichomonas vaginalis* (Aptima TV; Hologic Inc., San Diego, CA) assay were used according to the manufacturers’ instructions.

For *Candida* culture, vaginal ESwab specimens were collected and transported to the laboratory according to the manufacturer’s instructions and processed within 24–48 h of collection. BD BBL™ CHROMagar™ *Candida* medium (CHROMagar) and BD BBL™ Sabouraud Dextrose Agar, Emmons (SDA-Emmons) plates were inoculated with 100 μL of the liquid Amies medium. The CHROMagar plate was incubated at 33–37 °C and read after 36–48 h. The SDA-Emmons plate was incubated at 25–30 °C and read after 36–48 h and after 64–80 h of incubation. *Candida* isolates were identified using Bruker MALDI Biotyper®.

For Gram stain and Nugent score, a 50-μL aliquot of the vaginal ESwab specimen used for *Candida* culture was used to prepare a smear for Gram staining. Stained slides were evaluated by a single technologist for bacterial vaginosis using the Nugent score criteria (negative 0–3, indeterminate 4–6, positive ≥ 7) [17].

Chart review of patient clinical data was performed for all patients in the study to determine clinical signs and symptoms of vaginal infection: symptoms of vaginitis, urinary symptoms, abdominal symptoms, prior antibiotic or antifungal treatment, date and duration of therapy, other sexually transmitted infections, and clinician’s evaluation and diagnosis.

### Data analysis

Samples with discordant test results between MAX VP and Affirm were adjudicated by the following methods: *Candida* was confirmed using *Candida* culture, BV was confirmed using the Nugent score criteria, and *T. vaginalis* was confirmed with Aptima *Trichomonas vaginalis* assay. A composite reference standard was used to assess the performance of Affirm and MAX VP. A true positive was defined as a positive result for both MAX VP and Affirm or a positive test result from adjudication of discordant results by the Nugent score, Candida culture, or Aptima TV assay. Sensitivity, specificity, positive predictive value (PPV), negative predictive value (NPV), and overall percent agreement (OPA) were calculated for each target following comparison to the composite reference (which includes adjudication of discordant results). Fisher’s exact test was used to calculate the statistical significance of sensitivity and specificity.

## Results

### Subject population

Among the 200 compliant subjects, the age range was 18–77 years with a mean age of 30.5 years. Patients often presented with multiple symptoms, the most frequent of which included discharge (55%), itching (50%), and odor (43%). The average duration of symptoms was 19.7 days; 13 patients documented > 6 months of symptom duration (Table 1). When evaluating both Affirm and MAX VP, 20% (40/200 subjects) were negative for all targets tested. BV was the most prevalent condition detected (41.6%; 79/190), followed by *Candida* spp. (32.1%; 62/193) and *T. vaginalis* (4.2%; 8/192) (Table 2).

**Table 1:** Demographics for 200 compliant patients included in the study

Patient demographics		
Age	Mean	Median (range)
	30.5	29 (18–77)
Race/ethnicity	Patients	%
White	71	36
Non-White/Hispanic	78	39
White/Hispanic	17	9
Native American	14	7
Black/African American	8	4
Asian	6	3
Other/did not answer	6	3
Clinical symptoms	*N*	%
Discharge	110	55
Itching	99	50
Burning	56	28
Odor	86	43
Dysuria	27	14
Pyuria	9	5
Abdominal pain	15	8
Abdominal cramping	24	12
Other	25	13
Symptoms		
Symptoms per patient	Number of symptoms	*N* (%)
	Not documented	11 (5.5)
	1	49(24.5)
	2	65 (32.5)
	3	42 (21.0)
	4	19 (9.5)
	5	13 (6.5)
	6	1 (0.5)
Average symptoms/patient		2.1
Average duration of symptoms (days)		19.7
Other causes of symptoms		
	Patients	%
Chlamydia	3	1.5
Gonorrhea	1	0.5
Genital HSV	1	0.5
Urinary tract infection	13	6.5
Treatment based on standard-of-care result		
	Positive test result	Treated, *N* (%)
BV	95	87 (91.6)
VVC	43	32 (74.4)
TV	8	7 (87.5)

*HSV* herpes simplex virus, *BV* bacterial vaginosis, *VVC* vulvovaginal candidiasis, *TV Trichomonas vaginalis*

**Table 2:** Overall performance of BD MAX vaginal panel and BD Affirm VPIII assays

	Prevalence	TP	FP	TN	FN	Sensitivity		Specificity		PPV	NPV
						%	95% CI	%	95% CI	%	%
MAX VP-BV	41.6%^†^	76	4	99	3	96.2	89.3–99.2	96.1	89.8–98.7	95.0	97.1
Affirm-GV		76	19	84	3	96.2	89.5–99.2	81.6	72.7–88.5	80.0	96.6
MAX VP-*Candida*	32.1%	61	6	125	1	98.4	91.3–99.6	95.4	90.3–98.3	91.4	99.2
Affirm-*Candida*		43	0	131	19	69.4	56.4–80.4	100.0	97.2–100	100.0	87.3

Total percent positive agreement 97.3%, percent negative agreement 97.8%, percent overall agreement 97.7%; Affirm percent positive agreement 85.9%, percent negative agreement 95.5%, percent overall agreement 94.1%. *P* < 0.001 for the performance of MAX VP compared to the Affirm

*BV* bacterial vaginosis, *MAX VP* BD MAX Vaginal Panel, *TP* true positive, *FP* false positive, *TN* true negative, *FN* false negative, *PPV* positive predictive value, *NPV* negative predictive value

†8 specimens were indeterminate by Nugent score criteria

### Bacterial vaginosis

Patients positive for BV most frequently presented with symptoms of odor (55.7%; 44/79), discharge (55.7%; 44/79), and itching (46.8%; 437/79). When the duration of symptoms could be documented, BV-positive patients reported an average of 22.2 days of symptoms prior to seeking care; six patients reported > 6 months of symptom duration. MAX VP performed significantly better than Affirm with a specificity of 96.1% and only 5.1% (4/79) false-positive results (Table 2). The specificity of Affirm-GV was 81.6%. There were 29 Affirm-GV positive/MAX VP-BV negative samples. Of these patients, 96.6% (28/29) were treated for BV based on the positive Affirm-GV result (Table 3). Following discordant resolution of these samples by Nugent score criteria, only 10.3% (3/29) were true positive, 65.5% (19/29) were true negative, and 24.1% (7/29) were indeterminate (Table 3). All 19 patients with false-positive Affirm results were treated for BV based on these standard-of-care results and clinical presentation. Eight patients were MAX VP-BV positive/Affirm-GV negative; 50% (4/8) were true negative, 37.5% (3/8) were true positive, and 12.5% (1/8) were indeterminate by Nugent score criteria (Table 3). Of the MAX VP-BV positive patients with negative Affirm result, one patient was diagnosed and clinically treated for presumed BV despite the negative standard-of-care results. Two additional patients had a history of recurrent BV.

**Table 3:** Discordant analysis for the detection of bacterial vaginosis

	Nugent positive	Nugent negative	Nugent indeterminate	Treated for BV, *N* (%)
Affirm positive/ MAX VP negative	3	19	7	28/29 (96.5)
Affirm negative/ MAX VP positive	3	4	1	1/8 (12.5)

Discordant analysis for the detection of organisms associated with bacterial vaginosis or normal flora. Nugent scoring criteria were used to adjudicate discordant results. Nugent negative 0–3, indeterminate 4–6, positive ≥7

### *Candida* species

Patients who were positive for *Candida* spp. most commonly presented with itching (71.0%; 44/66), discharge (62.9%; 39/66), and burning (38.7%; 24/66). Patients with *Candida* spp. had symptoms for an average of 23.7 days prior to presenting to care, with an additional 4 having symptoms for greater than 6 months. The performance characteristics of Affirm and MAX VP for the detection of *Candida* spp. were evaluated. The Affirm-*Candida* result does not differentiate *C. albicans*, *C. glabrata*, *C. kefyr*, *C. krusei*, *C. parapsilosis*, and *C. tropicalis* while the MAX VP assay (MAX VP-*Candida*) differentiates *Candida* group, *C. glabrata*, and *C. krusei*. For the initial analysis, detection of *Candida* species for MAX VP-*Candida* was not differentiated by species and the results are presented in Table 2. Affirm-*Candida* had a sensitivity, specificity, NPV, and PPV of 69.4%, 87.3%, 100%, and 100%, respectively, compared to 98.4%, 99.2%, 95.4%, and 91.4% for MAX VP-*Candida* (Table 2). When *Candida* species were differentiated using MAX VP results, there were 63 *Candida* group, 9 *C. glabrata*, and 0 *C. krusei* detected (Table 4). For MAX VP-*Candida*, 88.9% (56/63) of the *Candida* group (55 *C. albicans* and 1 *C. dubliniensis*) were confirmed in culture for an overall percent agreement with culture of 95.9% (98.3% positive agreement and 94% negative agreement) (Table 4).

**Table 4:** Performance of BD MAX Vaginal Panel for detection of *Candida* spp. after discordant analysis

	*N*	TP	FP	TN	FN	PPA	NPA	Overall agreement
*Candida* group	63	56^†^	7^††^	129	1	98.3%	94.9%	95.9%
*Candida glabrata*	9	8	1^*^	184	0	100.0%	99.5%	99.5%

Performance of MAX VP for detection and differentiation of *Candida. Candida* group includes *C. albicans*, *C. tropicalis*, *C. parapsilosis, C. dubliniensis*, *C. glabrata*, and *C. krusei*

*TP* true positive, *FP* false positive, *TN* true negative, *FN* false negative, *PPA* positive percent agreement, *NPA* negative percent agreement

^†^Organisms recovered: 55 *C. albicans*, 1 *C. dubliniensis*

^††^Organisms recovered: 1 *C. glabrata*, 6 no *Candida* isolated

*Organisms recovered: 1 *C. albicans*

For *C. glabrata*, 88.9% (8/9) of the samples positive by MAX VP were confirmed by culture for an overall agreement of 99.5% (100% positive agreement, 99.5% negative agreement) with culture (Table 4). Of the patients who were positive for *Candida* using the standard-of-care Affirm test, eight had true-positive *C. glabrata* as determined by culture and MAX VP and six were treated with fluconazole based on the Affirm result.

### *Trichomonas*

The performance characteristics of Affirm and MAX VP for the detection of *Trichomonas vaginalis* were evaluated. There was 100% concordance between Affirm-TV and MAX VP-TV. Seven of the eight patients who were TV-positive by MAX VP were also positive for BV.

## Discussion

The results of this study demonstrate that MAX VP provides better specificity for the detection of BV compared to Affirm with no difference in sensitivity, whereas it provides a superior sensitivity, with specificity that is at least as good, when compared to Affirm for the detection of *Candida* spp.; an equivalent performance for TV in symptomatic patients was observed for MAX VP compared to Affirm.

The prevalence of BV in this study was 41.6%; other studies report a BV prevalence of about 20–64% in symptomatic women [15, 18]. Affirm had a specificity (81.6%) for BV that is consistent with previous reports [15, 19]. MAX VP was associated with a significantly better specificity of 96.1% compared to Affirm. The difference in specificity for these two assays is likely due to the detection of *G. vaginalis* alone in Affirm compared to the algorithmic detection of a combination of markers in the MAX VP assay. The inclusion of *Lactobacillus* spp. as an indicator of normal flora and the presence of *G. vaginalis*, *A. vaginae*, *Megasphaera-1*, and BVAB-2 as indicators of BV in MAX VP increase the specificity for the detection of BV. The sensitivity for both assays was identical (96.2%), with each having 3 false-negative results.

For VVC, 29.0% of infections were caused by *Candida* group and 4.1% of infections were caused by *C. glabrata*; there were no *C. krusei* detected in the study, and the overall *Candida* spp. prevalence was 32.1%. Overall, MAX VP significantly outperformed Affirm with a sensitivity of 98.4% compared to 69.4%. An additional 18 true-positive samples were detected using MAX VP. Although Affirm does not differentiate *Candida* spp., it detects *C. albicans*, *C. glabrata*, *C. kefyr*, *C. krusei*, *C. parapsilosis*, and *C. tropicalis*. Therefore, only the one sample containing *C. dubliniensis* would not have been detected in Affirm based on the absence of target coverage.

MAX VP differentiates *C. glabrata* and *C. krusei*, which are important causes of VVC, but clinically indistinguishable from *C. albicans*. Speciation of *Candida* for VVC may be important as 50% of *C. glabrata* isolates from VVC have decreased sensitivity to fluconazole and *C. krusei* is intrinsically resistant to the antifungal agent. Although optimal therapy for non-*albicans* VVC has not been identified, longer duration with a non-fluconazole azole regimen or use of a vaginal boric acid capsule is recommended [1]. *C. glabrata* and other non-*albicans Candida* species have also been recovered in 10–20% of women with recurrent VVC (RVVC), which is defined as four or more episodes of symptomatic VVC within 1 year [20]. The data for treatment of non-*albicans* VVC is likely limited due to the lack of testing and differentiation of non-*albicans* isolates. Increased recognition of the prevalence of non-*albicans* VVC and their role in RVVC may provide additional data to optimize treatment choices.

This study has some limitations. The patient sample size was relatively small which likely impacted the detection of TV and the assessment of differences between Affirm-TV and MAX VP-TV. Although TV infections were limited in this study population, it is important to detect this pathogen in the differential diagnosis and treatment of vaginitis since patient symptoms often overlap. Moreover, these findings are consistent with other studies showing that co-infections with TV and BV can occur [21], which underscore the need for accurate diagnosis. A larger study may be important to understand the prevalence of TV and the extent or impact of co-infections involving TV, BV, or *Candida* on treatment or patient management in this study population. While *C. glabrata* was identified in this study, antifungal susceptibility testing was not performed to determine if the efficacy of empiric fluconazole treatment would be impacted by the detection of this organism. Overall, when comparing the two commercially available assays for the most common causes of vaginitis, MAX VP had improved performance and diagnostic accuracy for the diagnosis of vaginitis.
